# Supplementary material for: Predictions for Three-Month Postoperative Vocal Recovery after Thyroid Surgery from Spectrograms with Deep Neural Network
Source: Sensors (Basel). 2022 Aug 24;22(17):6387. doi: 10.3390/s22176387 (PMC9460363; doi:10.3390/s22176387)
Supplement: Supplementary file 1 [file sensors-22-06387-s001.zip › SupplementaryMethods.pdf]

Original Article

Predictions for Three-Month Postoperative Vocal Recovery af-ter Thyroid Surgery from Spectrograms with Deep Neural Network

Running title: Deep learning predicts postoperative vocal recovery

Jeong Hoon Lee[1]*, Chang Yoon Lee[2]*, Jin Seop Eom[3], Mingun Pak[4], Hee Seok Jeong[5], Hee Young Son[2]

[1]Division of Biomedical Informatics, Seoul National University Biomedical Informatics (SNUBI), Seoul National University College of Medicine, Seoul, 110799, Republic of Korea,

[2]Department of Otolaryngology, Thyroid/Head & Neck Cancer Center of The Dongnam Institute of Radiological & Medical Sciences (DIRAMS), Busan, Korea,

[3]Samsung Electronics Co., Ltd.

[4]IBM Silicon Valley Lab, San Jose, California, United States of America

[5]Department of Radiology, Pusan National University Yangsan Hospital, Yangsan, Korea

*Contributed equally

Corresponding Author

Hee Young Son, M.D. Department of Otolaryngology, Thyroid/Head & Neck Cancer Center of The Dongnam Institute of Radiological & Medical Sciences (DIRAMS), Busan, Korea

Phone: +82-51-720-5287; Fax: +82-51-5914; E-mail: hyson79@gmail.com

Keyword: Deep-learning, Voice recovery, Spectrogram

Supplementary Methods

The overall structure of the proposed method for predicting GRBAS score after surgery

The structure below is a simple representation of the deep learning model used in this study to predict GRBAS 3 months after surgery. We used Keras deep learning framework included in tensorflow 2.3.0 version. We trained the model using the Adam optimizer to accelerate the convergence of network parameters with learning rate 0.001. The learning rate was gradually reduced through the call-back function.

Layer (type)	Output Shape
EfficientNetB4_A (InputCNN_A)	(None, 224, 224, 3)]
EfficientNetB4_B (InputCNN_B)	(None, 224, 224, 3)]
Model_A-Pooling (GlobalMaxPooling)	(None, 1792)
Model_B-Pooling (GlobalMaxPooling)	(None, 1792)
dense_A (Dense)	(None, 1024)
dense_B (Dense)	(None, 1024)
reshape (Reshape)	(None, 1, 1024)
reshape_1 (Reshape)	(None, 1, 1024)
concatenate (Concatenate)	(None, 2, 1024)
LSTMLayer (LSTM)	(None, 2, 1024)
GRBAS (Dense)	(None, 2, 5)

Total params: 43,227,327

Preprocessing of the spectrogram from patient's voice

```
from python_speech_features import mfcc, delta, logfbank
from scipy import signal
import scipy.io.wavfile as wav
import matplotlib.pyplot as plt
import os
import soundfile as sf
import numpy as np

import numpy as np
from matplotlib import pyplot as plt
import scipy.io.wavfile as wav
from numpy.lib import stride_tricks
```

```
from matplotlib import cm


def stft(sig, frameSize, overlapFac=0.5, window=np.hanning):
    win = window(frameSize)
    hopSize = int(frameSize - np.floor(overlapFac * frameSize))

    # zeros at beginning (thus center of 1st window should be for sample nr. 0)
    samples = np.append(np.zeros(int(np.floor(frameSize/2.0))), sig)
    # cols for windowing
    cols = np.ceil( (len(samples) - frameSize) / float(hopSize)) + 1
    # zeros at end (thus samples can be fully covered by frames)
    samples = np.append(samples, np.zeros(frameSize))

    frames = stride_tricks.as_strided(samples, shape=(int(cols), frameSize),
strides=(samples.strides[0]*hopSize, samples.strides[0])).copy()
    frames *= win
    print("frames: ", frames)

    return np.fft.rfft(frames)


def logscale_spec(spec, sr=44100, factor=20.):
    timebins, freqbins = np.shape(spec)

    scale = np.linspace(0, 1, freqbins) ** factor
    scale *= (freqbins-1)/max(scale)
    scale = np.unique(np.round(scale))

    # create spectrogram with new freq bins
    newspec = np.complex128(np.zeros([timebins, len(scale)]))
    print("timebins: ", timebins)
    print("scale: ", scale)
    for i in range(0, len(scale)):
        if i == len(scale)-1:
            newspec[:,i] = np.sum(spec[:,int(scale[i]):], axis=1)
        else:
            newspec[:,i] = np.sum(spec[:,int(scale[i]):int(scale[i+1])], axis=1)

    print("freqbins: ", freqbins)
    # list center freq of bins
    allfreqs = np.abs(np.fft.fftfreq(freqbins*2, 1./sr)[:freqbins+1])
    freqs = []
    for i in range(0, len(scale)):
        if i == len(scale)-1:
            freqs += [np.mean(allfreqs[int(scale[i]):])]
        else:
            freqs += [np.mean(allfreqs[int(scale[i]):int(scale[i+1])])]
```

```
    return newspec, freqs


def plotstft(audiopath, binsize=2**10, plotpath='none', colormap=cm.jet):
    samplerate, samples = wav.read(audiopath)
    #samples, samplerate = sf.read(audiopath)
    print("sample rate: ", samplerate)
    s = stft(samples, binsize)

    sshow, freq = logscale_spec(s, factor=1.0, sr=samplerate)

    ims = 20.*np.log10(np.abs(sshow)/10e-6) # amplitude to decibel

    timebins, freqbins = np.shape(ims)

    #print("timebins: ", timebins)
    #print("freqbins: ", freqbins)

    plt.figure(figsize=(9,4.5))
    plt.imshow(np.transpose(ims), origin="lower", aspect="auto", cmap=colormap, interpolation="none")

    plt.xlabel("time (s)")
    plt.ylabel("frequency (hz)")
    plt.xlim([0, timebins-1])
    plt.ylim([0, freqbins])

    xlocs = np.float32(np.linspace(0, timebins-1, 5))
    plt.xticks(xlocs, ["%.02f" % l for l in ((xlocs*len(samples)/timebins)+(0.5*binsize))/samplerate])
    ylocs = np.int16(np.round(np.linspace(0, freqbins-1, 10)))
    plt.yticks(ylocs, ["%.02f" % freq[i] for i in ylocs])

    if plotpath:
        plt.savefig(plotpath, bbox_inches="tight")
    else:
        plt.show()

    plt.clf()

    return ims
```
