# Supplementary material for: Predictions for Three-Month Postoperative Vocal Recovery after Thyroid Surgery from Spectrograms with Deep Neural Network
Source: Sensors (Basel). 2022 Aug 24;22(17):6387. doi: 10.3390/s22176387 (PMC9460363; doi:10.3390/s22176387)
Supplement: Supplementary file 1 [file sensors-22-06387-s001.zip › SupplementaryTables.pdf]

Original Article

Predictions for Three-Month Postoperative Vocal Recovery af-ter Thyroid Surgery from Spectrograms with Deep Neural Network

Running title: Deep learning predicts postoperative vocal recovery

Jeong Hoon Lee[1]*, Chang Yoon Lee[2]*, Jin Seop Eom[3], Mingun Pak[4], Hee Seok Jeong[5], Hee Young Son[2]

[1]Division of Biomedical Informatics, Seoul National University Biomedical Informatics (SNUBI), Seoul National University College of Medicine, Seoul, 110799, Republic of Korea,

[2]Department of Otolaryngology, Thyroid/Head & Neck Cancer Center of The Dongnam Institute of Radiological & Medical Sciences (DIRAMS), Busan, Korea,

[3]Samsung Electronics Co., Ltd.

[4]IBM Silicon Valley Lab, San Jose, California, United States of America

[5]Department of Radiology, Pusan National University Yangsan Hospital, Yangsan, Korea

*Contributed equally

Corresponding Author

Hee Young Son, M.D. Department of Otolaryngology, Thyroid/Head & Neck Cancer Center of The Dongnam Institute of Radiological & Medical Sciences (DIRAMS), Busan, Korea

Phone: +82-51-720-5287; Fax: +82-51-5914; E-mail: hyson79@gmail.com

Keyword: Deep-learning, Voice recovery, Spectrogram

Supplementary Table S1. RMSE value for prognosis prediction according to the architecture of CNN model.

Model Architecture	G	R	B	A	S	Mean
EfficientNet-B4	0.399	0.365	0.409	0.469	0.203	0.369
NasNetV2	0.455	0.381	0.461	0.538	0.117	0.390
VGG19	0.537	0.364	0.558	0.588	0.108	0.431
DenseNet121	0.648	0.329	0.658	0.529	0.066	0.446
ResNet50	0.561	0.369	0.533	0.637	0.164	0.453
InceptionResNetV2	0.633	0.384	0.626	0.675	0.146	0.493
InceptionV3	0.748	0.364	0.773	0.757	0.317	0.592

Supplementary Table S2. Distribution of GRBAS scores in patients 6 months after surgery

Score	0	1	2
G	3	6	3
R	7	4	1
B	4	6	2
A	6	5	1
S	11	1	0
